# Flavonol Profile Is a Reliable Indicator to Assess Canopy Architecture and the Exposure of Red Wine Grapes to Solar Radiation

**DOI:** 10.3389/fpls.2019.00010

**Published:** 2019-01-31

**Authors:** Johann Martínez-Lüscher, Luca Brillante, Sahap Kaan Kurtural

**Affiliations:** ^1^Department of Viticulture and Enology, University of California, Davis, Davis, CA, United States; ^2^Department of Viticulture and Enology, California State University, Fresno, Fresno, CA, United States

**Keywords:** flavonoids, solar radiation, temperature, water status, fruit ripening, grape composition, precision agriculture, UV-B radiation

## Abstract

Exposure to solar radiation is a determining factor of grape composition. Flavonol synthesis is upregulated by solar radiation leaving a fingerprint on flavonol profile. This study aimed to test the factors affecting flavonol accumulation and profile and their potential as an indicator to assess the overall exposure of red wine grape berry to solar radiation. We performed three experiments to study the response of flavonol accumulation and profile to (1) three different solar radiation exclusion treatments during berry development; (2) canopy porosity and leaf area index (LAI); and (3) spatial variability of water status, vigor and ripening and cultural practices in commercial vineyards. Results showed a strong relationship between global radiation, inverse dormant pruning weights or canopy porosity (inversely proportional to LAI) and % kaempferol or % quercetin. Furthermore, the increase in concentration of the above two flavonols was associated with a reduction of % myricetin. Total flavonol content, % kaempferol, % quercetin, and % myricetin had significant correlations with inverse dormant pruning weights, but these were less sensitive to over-ripening or water deficits. Flavonol profile was associated to site hydrology (wetness index) through changes in vigor, and to LAI; and responded to shoot thinning or fruit-zone leaf removal. These results support the reliability of the flavonol profile as an assessment parameter for studies aiming to discuss canopy architecture or the effect of solar radiation on grapevine berries.

## Introduction

Flavonols are mainly accumulated in epidermal cells of plant tissues in response to solar radiation, especially UV-B, filtering the most harmful part of the solar spectrum to DNA (Kolb et al., 2001). Flavonols are known to play a role complementary to xanthophylls, protecting the photosynthetic apparatus *in situ* from excess of solar radiation (Agati et al., 2013; Castagna et al., 2017). Due to their strong radical scavenging activity, flavonoids such as flavonols and anthocyanins are involved in the mitigation of drought-related oxidative damage (Nakabayashi et al., 2014). In a wider context, flavonoids are one of the most versatile secondary metabolites in plants. For instance, they play a key role promoting sexual reproduction of plants, enabling a normal development of pollen and making flowers noticeable to pollinators or fruits to animals that contribute to seed dispersal (Koes et al., 1994). They also play an important role in cross-talk between plants and microorganisms, as they are released as exudates into the rhizosphere (Cooper, 2004). Flavonoids are also remarkable for their inhibitory activity on inter-species seed germination and on fungal pathogens (Kong et al., 2004).

Within each class of flavonoids, structural variation exists in their basic 15-carbon skeleton, leading to different physicochemical properties. Anthocyanins are colorful molecules and hydroxyl and methyl group substituents in positions 3′ and 5′ of the B-ring give place to six kind of compounds (Pelargonidins, Cyanidins, Peonidins, Delphinidins, Petunidins, and Malvidins), which can give place to a range of colors from orange to purple to plant tissues (Dixon et al., 2013). Although flavonols are practically colorless, their substituents in the B-ring may also lead to different optical properties appreciable in the absorbance in the UV range (Csepregi and Hideg, 2018). For both anthocyanins and flavonols, there is a relationship between the substituents in these positions and the chemical behavior. The addition of hydroxyl groups in positions 3′ and 5′ of the B-ring result in a great increase in their antioxidant capacity (Csepregi and Hideg, 2018).

**FIGURE 1:**
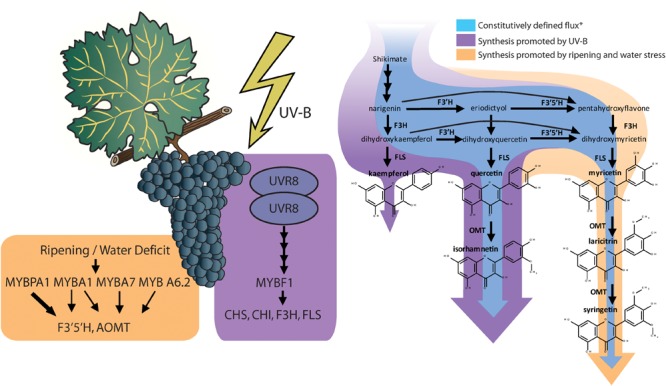
Induction of transcription factors and the structural genes they up regulate relevant to flavonol biosynthesis and diversification (reviewed by Matus, 2016; Matus et al., 2017; Left). Changes in flavonol synthesis observed by Martínez-Lüscher et al. (2014a) in response to supplemental monochromatic UV-B radiation and to water deficit under controlled conditions (Right). ^∗^Constitutive synthesis is deduced from the flavonol content of *V. vinifera* cv. Tempranillo grapes under UV-B filtered solar radiation, 24/14°C cycle (day/night) and pots watered to field capacity or cyclic drought. *Flavonol synthase* (*FLS*) is strongly upregulated by UV-B altering the balance with the levels of *flavonoid hydroxylases* (*F3*′*H* and *F3*′*5*′*H*), leading to a higher relative abundance of the less hydroxylated forms (kaempferol and quercetin and isorhamnetin).

Red and black-skinned grape varieties constitutively present a wide range of anthocyanin and flavonol profiles depending their substituents in 3′ and 5′ position, and interestingly, these do not necessarily correlate with each other (Mattivi et al., 2006). Most grapes used in medium and full-bodied red wines have a highly 3′ and 5′ substituted profile (rich in anthocyanins and flavonols with hydroxyl or methyl groups in 3′ and 5′ position of the B-ring). For instance, the cultivars Cabernet Sauvignon, Syrah, or Tempranillo being examples of highly 3′ 5′ substituted profiles and cultivars Sangiovese and Pinot Noir are examples of poorly 3′ 5′ substituted profiles, similar to most table grapes (Mattivi et al., 2006). Anthocyanin and flavonol profiles are highly determined genetically, controlled and inherited as a quantitative loci trait (Fournier-Level et al., 2011; Malacarne et al., 2015). In fact, flavonol profiles were proposed as an authentication tool for wines (Hermosín-Gutiérrez et al., 2011). Ultimately, anthocyanins and flavonol profiles are determined by the proportion in the levels of expression of the gene/s encoding flavonoid 3′ 5′ hydroxylases (F3′5′H) and the synthetic enzymes controlling the synthesis of each flavonoid, e.g., UFGT for anthocyanins and FLS for flavonols (Castellarin and Di Gaspero, 2007; Martínez-Lüscher et al., 2014a). In fact, there is also a strong environmental regulation of this trait. Water deficits and speed of sugar accumulation in the grape berry are involved in the upregulation of *F3*′*5*′*H* (Castellarin et al., 2007; Dai et al., 2014), encoding for the enzyme responsible for the hydroxylation of dihydrokaempferol and dihydroquercetin into dihydromyricetin (Kaltenbach et al., 1999), leading to higher proportion of hydroxylated anthocyanins and flavonols under water deficit (Castellarin et al., 2007; Martínez-Lüscher et al., 2014a). F3′5′Hs are actually a family of enzymes, with similar function and regulation (Falginella et al., 2010). This family of enzymes are controlled by two transcription factors; MYBA1, which is also involved in the up-regulation of downstream anthocyanin biosynthesis genes (e.g., *UFGT*), but specially MYBPA1, which is not involved (Matus et al., 2017; Figure 1).

The synthesis of flavonols has one master transcription factor, MYBF1, that controls the expression of *chalcone synthase (CHS), chalcone isomerase (CHI), flavone 3’ hydroxylase (F3*′*H)*, and *flavonol synthase (FLS)* (Czemmel et al., 2009). In turn, the expression of *MYBF1* is controlled through the signaling cascade derived from the photoreception of UV-B radiation by Ultraviolet Resistance Locus 8 (UVR8) homodimers (Matus, 2016; Figure 1). In previous research, Martínez-Lüscher et al. (2014b) reported that flavonol content in grape berry skins increased linearly with the exposure time to an artificial source of UV-B. This response corresponded also to a decrease in the proportion of tri-substituted flavonols (myricetin, laricitin, and syringetin glycosides) and an increase in the proportion of disubstituted flavonols (quercetin and isorhamnetin glycosides) and monosubstituted flavonols (kaempferol glycosides). The study of the interactive effects of UV-B with water deficits revealed that UV-B did not upregulates all genes involved in flavonoid biosynthesis in the same way (Martínez-Lüscher et al., 2014a). Whereas genes encoding for enzymes in upstream in the pathway may present a nearly twofold induction (e.g., *CHS* and *F3*′*H*), the induction of *FLS* by a high UV-B dose was 10-fold. This disproportion putatively resulted in stronger competition of FLS with flavonoid hydroxylases (F3′H and F3′5′H) for flavonol substrates, thus reducing their chances of being hydroxylated before being transformed into flavonols (Figure 1) and leading to a lower hydroxylation profile in flavonols but not anthocyanins.

In grape production, canopy density is controlled either during the dormant period through pruning, or during the growing season through leaf removal and shoot thinning. These practices result in higher canopy porosity leading to enhanced ripening, air circulation and exposure of grapes to solar radiation. Fruit exposure has been associated to a reduction in humidity and fungal diseases (English et al., 1993), and some desirable effects for specific winemaking targets, such as herbaceous aroma removal (Koch et al., 2012) and higher content of flavonoid compounds in berries (Matus et al., 2009). Conversely, the impact of the excess of solar radiation can be deleterious, resulting in damage to grape berry and a decrease in berry flavonoid content and acidity (Martínez-Lüscher et al., 2017). Therefore, there is a strong need of assessing canopy porosity and the exposure of red grapes to solar radiation. The aim of this study was to deduce the relationship between flavonol profile and solar radiation under different field conditions and the use of flavonol profile in the assessment of parameters associated with canopy architecture and grape berry microclimate.

## Materials and Methods

### Experiment 1: Response of Flavonol Profile Development Under 3 Solar Radiation Exclusion Treatments

An experiment was conducted in 2016 in a vineyard in Oakville, CA. Plants were 7-year-old *Vitis vinifera* cv. Cabernet Sauvignon clone FPS08 (Foundation Plant Services, University of California, Davis, United States) grafted into 110R (*Vitis rupestris* × *Vitis berlandieri*) spur pruned with a bilateral cordon, shoots vertically positioned and a vine spacing of 2.4 m × 2 m (row × vine). A control (uncovered; 0% shade factor) and two polyethylene nets treatments (20% shade factor and 40% shade factor; Ginegar, Kibbutz, Israel) were installed on 27 May (31 days after anthesis). There were four treatment replicates consisted of three vines each. The uniformity of the nets’ light transmittance throughout the solar spectrum was verified with a spectrometer (Black Comet-SR, StellarNet; Tampa, FL, United States). In the range of 300 to 1000 nm, the minimal transmittance was from 77 to 58%, the maximal was 84 and 61%, and the average 81 and 60% for the 20 and 40% shading factors, respectively. On dates 20 June, 19 July, 29 July, 9 August, 19 August, 29 August, and 9 September, 55-berry samples were collected to determine TSS and 20-berry samples were collected, weighed and stored at -80°C for later analyses of flavonols through HPLC-DAD.

### Experiment 2: Relationship Between Variability in Canopy Porosity, LAI and Global Radiation and Flavonol Content and Profile

The experiment was conducted in 2017 in Oakville, CA (38.428° N, 122.409° W, 47 m asl) with row orientation NE-SW. Four rows of *V. vinifera* Cabernet Sauvignon clone FPS08 grafted onto 420A (*Vitis riparia* × *V. berlandieri*) with bilateral cordon and vine spacing of 2 m × 2 m (row × vine). In order to induce some variability cluster exposure, eight rows were pruned as (1) 2-bud spurs positioning using two sets of wires 0.3 and 0.6 m from the cordon to keep shoots growing upward between the wires in a single plane (i.e., vertical-shoot-positioned trellis); and (2) cane-pruned leaving 0.5 m-long fruiting canes and letting shoots sprawl supported by the theriomorphism of shoot tendril on the two sets of wires (i.e., similar to California sprawl). For each of the 8 random grapevines used (4 for each of the training systems), 2 clusters facing either east, interior or west (48 clusters in total) were flagged. Images from the cluster perspective were captured at two times (24 of August and 9 September) using a 150° hemispherical lens coupled to a smartphone (iPhone SE, Apple, Cupertino, CA, United States). Although 180° hemispherical lens are ideal, 150° lens have been successfully tested canopy analyses (Bianchi et al., 2017). In addition, the distance between rows and the neighbor plants’ canopy height (ca. 1.2 m) contribute to reduce the error of assuming the 15° angle omission on each side being blocked for the calculation of global radiation. The images were processed in R (version 3.2.5-6). Thus, a simple thresholding condition was applied in the same way to all images blue channel to convert them into binary pixels (black/white). Canopy porosity was then calculated as the % of binary pixels capturing the sky. Leaf area index (LAI), which in this case accounted also for non-leafy vegetation, was estimated using the principles of commercial canopy analyzers (Welles and Norman, 1991) adapted to hemispherical images by ter Steege (1997). In brief, mean canopy gaps fraction of five 13° wide angular bands at five zenith angles (7, 23, 38, 53, and 68°) were calculated and weighed as 0.034, 0.104, 0.160, 0.218, 0.494, respectively. Global radiation was calculated simulating the trajectory of the sun through the period from beginning of ripening (24 July) to harvest (9 September). We assumed that diffuse radiation was 15% of the direct radiation under clear sky conditions, modulated by canopy porosity but not cloud cover. Modeled direct radiation values were corrected with the onsite CIMIS meteorological station actual data before summing accumulated radiation (Supplementary Information 1). On 9 September, 5-berry samples were collected from the top of each cluster. Grape berries were weighed and skinned. Total soluble solids (TSS) were determined from the pulp juice and skins were collected and stored at -80°C for later analyses of anthocyanins and flavonols through HPLC-DAD. To characterize the maximum temperature gain of clusters under such conditions, on September 11, 2017, temperature of 4 fully exposed clusters from each side of the row (facing SE and NW) and four plants with vertical-shoot positioning trellis were determined with an infra-red thermometers (Spectrum technologies; Aurora, IL, United States).

### Experiment 3: Relationship Between Flavonol Profile and Natural and Induced Variability in Commercial Vineyards

Two commercial vineyards were monitored during 2016 season in CA, United States. In Healdsburg, CA (38.66° N, 122.91° W, 32 asl), we used a 3.5 ha vineyard of 19-year-old Cabernet Sauvignon grafted onto 110R (*V. berlandieri* × *V. rupestris*) and a vine spacing of 2.14 m × 3.35 m (vine × row). Trellis consisted of a double bilateral cordon (as in a Lyre trellis) with spurs pruned to 2 buds per spur, and shoots trained upward by a set of wires at 0.3 m from the cordon, letting shoots bend downward and to the sides by its own weight. In Paso Robles, CA (35.58° N, 120.63° W, 223 asl), we used a 6 ha vineyard of 14-year-old Merlot grafted onto 1103P (*V. berlandieri* × *V. rupestris*) and a vine spacing of 1.83 m × 2.44 m (vine × row). Trellis consisted in a bilateral cordon pruned to 2-bud spurs positioning using two sets of wires 0.3 and 0.6 m from the cordon to keep shoots growing upward between the wires in a single plane. Within these vineyards, experimental units consisting in 5 plants each were spread through each plot in a regularly spaced grid (33 and 40 m between the units, respectively). In Paso Robles Merlot vineyard, in order to emulate the most common industry practices, fruit-zone leaf removal on the North side, shoot thinning down to 25 shoots per plant (13.6 per meter), and combination of leaf removal and shoot thinning were applied randomly on 27 July to 12 randomly distributed experimental units in addition to the plants on the regularly spaced-grid. When maturity was reached, 55-berry samples were collected to determine TSS and 20-berry samples were collected, weight and stored at -80°C for later analyses of flavonols through HPLC-DAD.

### Plant Water Status

Plant water status was assessed by means of stem water potential (Ψ_stem_) measured after solar noon (13–15:00). Three to five leaves from each experimental unit were covered with a zip-top plastic bag and aluminum foil for 2 h prior to measurement. Leaves were excised with a razor blade and Ψ_stem_ was determined using a Scholander-type pressure bomb (model 615, PMS, Albany OR, United States). There were seven and four plant water status measurement dates collected for Healdsburg and Paso Robles vineyards, respectively. Whereas for Healdsburg Cabernet Sauvignon vineyard all the experimental units arranged in a grid sampling were measured (*n* = 35), for Paso Robles Merlot, only 24 experimental units were measured, 12 of these being subjected to leaf removal, shoot thinning or both.

### Canopy Size

Canopy size was estimated using dormant pruning wood weight. Although dormant pruning wood does not include leaves, it is a quick and reliable indicator of canopy size used in viticulture. In January 2017, previous year’s growth was pruned and individually weighed in all the experiments. For easier comparisons between trellis, dormant pruning weights are expressed in kg per meter of row (kg/m). The transmission of solar radiation through canopies necessarily follows an inverse proportionality relationship as dormant pruning weights increased. The inversion of dormant pruning weights resulted into a linear positive relationship with light transmission (Supplementary Information 2), helping to interpret their relationship with flavonols.

### Spatial Data Acquisition

A high-resolution digital elevation model (DEM) was acquired using a differentially corrected GPS (post processing accuracy was 2–5 cm in all x, y, z directions), TRIMBLE Pro 6T DGNSS receiver (Trimble Inc., CA, United States), and used for terrain analysis using the routines in SAGA GIS (Conrad et al., 2015) as in Brillante et al. (2017). Normalized vegetation difference index (NDVI) was obtained from a 4 band PlanetScope Scene satellite image, with a 3 m resolution, satellite ID 0c59. The image was obtained around the beginning of ripening, the 2016-07-14 at 9:54, with 2.9° off-nadir angle, 45.6° sun elevation and 95° sun azimuth. The image was further registered, and topographically corrected using the cosine method on the previously described DEM (Riaño et al., 2003).

### Berry Skin HPLC Analyses

**FIGURE 2:**
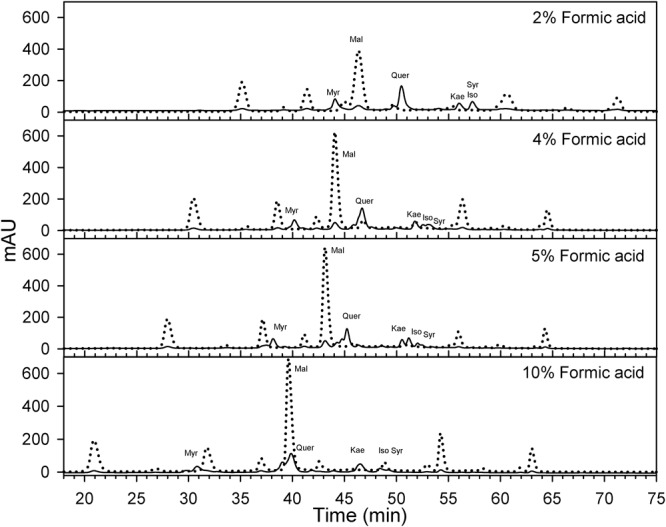
Effect of formic acid concentration on the optimization of HPLC method for the quantification of flavonols (λ = 365 nm; solid) in Cabernet sauvignon grape skins. A concentration of 5% of formic acid in the mobile phases allows the separation of different flavonols while avoiding the co elution with anthocyanins (λ = 520 nm; dotted) with the apparatus and column used in this study. Mal, malvidin-3-O-glucoside; Myr, myricetin-3-O-glucoside; Que, quercetin-3-O-glucoside; Kae, kaempferol-3-O-glucoside; Iso, isorhamnetin-3-O-glucoside; Syr, syringetin-3-O-glucoside.

Berry skins were freeze-dried (Cold Trap 7385020, Labconco, Kansas City, MO, United States). Dried tissues were ground with a tissue lyser (MM400, Retsch, Germany). Fifty mg of the powder were extracted with methanol: water: 7 M hydrochloric acid (70:29:1, V:V:V) to determine flavonol concentration and profile. Extracts were filtered (0.45 μm, Thermo Fisher Scientific, San Jose, CA, United States) and analyzed using reversed-phase high performance liquid chromatography (HPLC) coupled to a diode array detector (DAD). The HPLC system was an Agilent 1260 series (Agilent, Santa Clara, CA, United States) with a reversed-phase C_18_ column LiChrospher^®^ 100, 250 mm × 4 mm with a 5 μm particle size and a 4 mm guard column of the same material. Anthocyanins may interfere significantly with the quantification of flavonols. Anthocyanin removal through solid phase extraction using a cationic exchange resin (e.g., Dowex 50X4-400, Acros Organics, Fair Lawn, NJ, United States) has been proposed for the determination of flavonols (Hilbert et al., 2015). However, the determination of flavonols is also possible avoiding co-elution between anthocyanins and flavonols (Downey and Rochfort, 2008). As Downey and Rochfort (2008) method was not possible to implement directly on our HPLC system, the method was fine-tunned for our instruments. Flow was set to 0.5 ml per minute and temperature was set to 25°C. Two mobile phases were designed to always maintain the following proportions (V/V) of acetonitrile, 0–8 min 8%, at 25 min 12.2%, at 35 min 16.9%, at 70 min 35.7%, 70–75 min 65%, and 80–90 min 8%. This acetonitrile gradient and different isocratic concentrations of formic acid (HCOOH) from 1.8 to 10% were tested by adjusting the gradients and concentrations of two mobile phases (aqueous HCOOH and HCOOH in acetonitrile) as in Supplementary Information 3. A concentration 5% of HCOOH was the only one, avoiding coelution and allowing the simultaneous quantification (Figure 2 and Supplementary Information 4). The remaining volume up to 100% was achieved with purified water. For our HPLC system and column, a 5% HCOOH helped to avoid co-elution, separation of individual flavonols and a high degree of peak sharpness in both anthocyanins and flavonols.

For the identification of flavonols, standards of myricetin-3-O-glucoside, quercetin-3-O-galactoside, quercetin-3-O-glucuronide, quercetin-3-O-glucoside, kaempferol-3-O-glucoside, isorhamnetin-3-O-glucoside and syringetin-3-O-glucoside (Extrasynthese, Genay, France) were used. Flavonols were quantified determining the peak area of the absorbance at 365 nm. Quercetin-3-O-glucoside was used as a quantitative standard for all the flavonols. It must be noted that each individual anthocyanin and flavonol have a different molar relative response factors (e.g., absorbance per M unit) and even though calculating a response factors for each flavonol would have been possible using commercial standards, this is not the standard practice in the literature and would make comparisons of flavonol profiles harder.

### Statistical Analyses

Linear regression was performed with Sigmaplot 13.0 (Systat Software, San Jose, CA, United States). Pearson’s correlation analyses were performed. Segmented regression was used to determine the breaking point in the relationship between global solar radiation, flavonol content and flavonol profile with “segmented” 0.5-0.3 R package (Muggeo, 2008). For data arranged by categorical factors (treatments), ANOVA combined with a LSD *post hoc* was run using “agricolae” 1.2-8 R package (de Mendiburu, 2016). Geostatistical analysis and kriging were performed using the “gstat” 1.1-4 R package (Pebesma, 2004). Because of marked trends with elevation or latitude/longitude, which are potentially related to soil variability, universal block kriging was used to interpolate % kaempferol and NDVI. Variogram shape was assessed by multiple tests and comparisons using cross-validation, please refer to Brillante et al. (2017) for further information. Modified *t*-test for Pearson’s correlation was applied in spatially distributed data to correct for spatial trends effect with “SpatialPack” 0.2-3 R package (Osorio et al., 2014).

## Results

### Effect of Shading Factor on Flavonol Profile Throughout Ripening and Over Ripening

**FIGURE 3:**
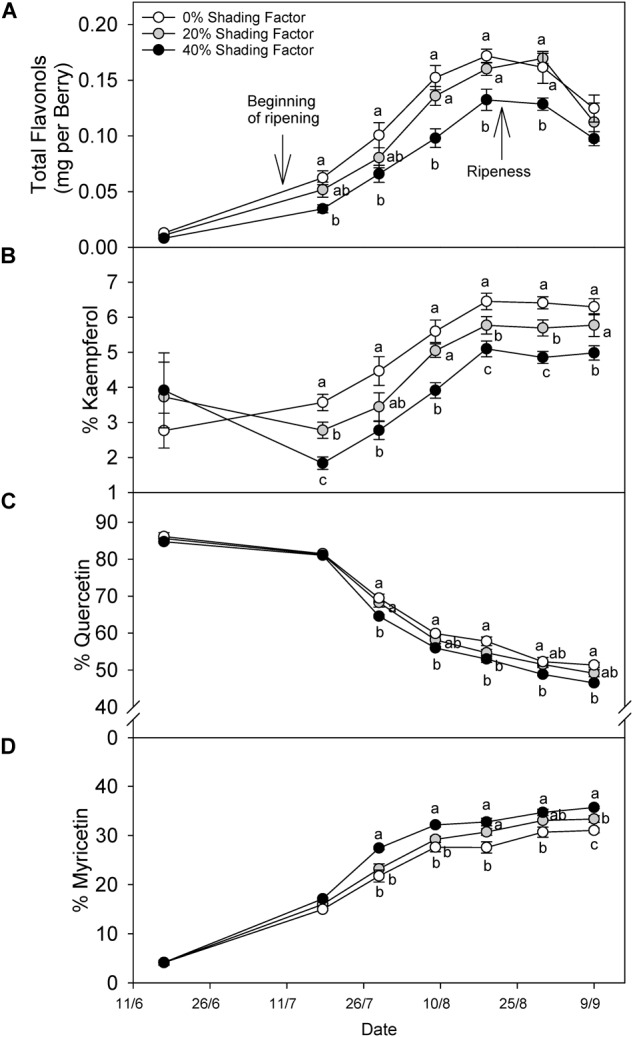
Evolution of flavonol content per berry **(A)**, % kaempferol **(B)**, % quercetin **(C)**, and % myricetin **(D)** under ambient (0% shading factor) and under two shade nets (20 and 40% shading factor) covering the fruit-zone of cv. Cabernet Sauvignon grapes. Ripening is considered from color change (ca. 12°Bx) to soluble solids of ca. 22°Bx and over-ripening from 23°Bx to harvest; 25°Bx in this case. Means in the same time point with no letters in common differ (ANOVA-LSD, *p* < 0.05).

Grapes grown under a shading net had lower flavonol content throughout ripening (Figure 3A). The content difference declined after ripening, and in fact, this was not significantly different at harvest. Changes in flavonol profile displayed different patterns among the three major groups of flavonols. For instance, % kaempferol (Figure 3B) increased throughout ripening under all shading conditions until 19 August (20.9°Brix) and remained similar through 30 August (22.5°Brix) and 9 September (24.5°Brix). The proportion of kaempferol was not different before veraison but during ripening the significant trend 0% > 20% > 40% shading factor appeared. The proportion of quercetin (Figure 3C) and myricetin (Figure 3D) flavonols combined was always more than 80% of total flavonols, and therefore, the increase in the proportion of one, was accompanied by a decrease in the other. The proportion of Quercetin decreased in all the treatments until harvest and increasing shading factors resulted in lower quercetin proportions. Contrarily, we measured the opposite response for % myricetin.

### Response of Flavonol Content and Profile to Modeled Global Radiation and Contribution of Flavonol Degradation to the Profile

**FIGURE 4:**
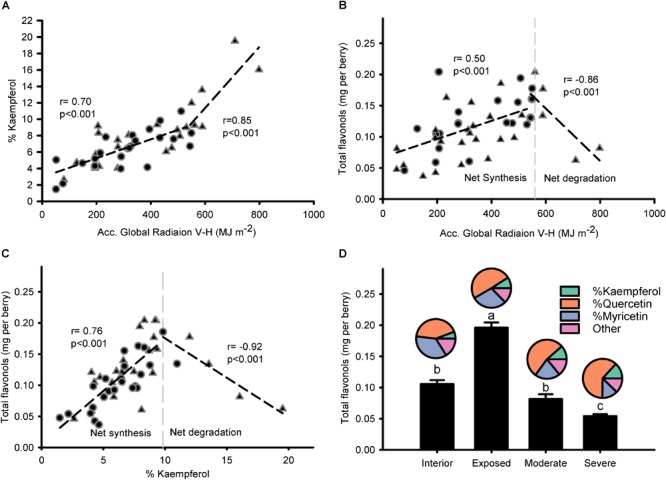
Flavonol profile is intimately related to the accumulated global radiation received by grapes and this response is exacerbated when degradation takes place. Correlation between accumulated global radiation from beginning of ripening to maturity and % kaempferol **(A)** or total flavonols per berry **(B)**. Correlation between % kaempferol and total flavonols per berry **(C)**. Berry flavonol content (bars) and profile (pies) of grapes from interior, exposed, moderately, and severely overexposed clusters **(D)**. Circles for sprawling canopy and triangles for vertical-shoot-positioned trellis. Dashed lines are breaking points determined through segmented regression.

Modeled accumulated radiation was strongly correlated to % kaempferol (Figure 4A), with a stronger gradient for higher doses of global radiation. Breaking point for the relationship between global radiation and % kaempferol was found at 544.2 MJ m^-2^; similar to the relationship between global radiation and total flavonols, which had a breaking point at 560.2 MJ m^-2^. Thus, two phases were visible in the response of flavonols to solar radiation: A first one, where flavonols increased (i.e., net synthesis) up to approximately 550 MJ m^-2^ from beginning of ripening to harvest (11.7 MJ m^-2^ per day); and a second one, where total flavonols decreased abruptly with doses above 550 MJ m^-2^ (Figure 4B). This relationship was sharper when % kaempferol was used as an indicator of solar radiation received by the grapes (Figure 4C). The temperature of fully exposed clusters in this vineyard measured on 11 September reached a maximum average temperature of 46.5°C, 15.4°C above air temperature (Supplementary Information 5). These results were supported by the higher content of flavonols in exposed clusters and the abrupt decrease in those grapes with visual symptoms of overexposure (Figure 4D). Despite these changes in concentration, flavonol profile only changed in one direction as exposure increased; increasing % kaempferol and % quercetin in detriment to % myricetin. The rest of the flavonol profile was constituted by methylated flavonols that remained constant ca. 18% from interior through overexposed grapes.

### Relationship Between Flavonol Content and Profile to Predict With Canopy Porosity and Leaf Area Index (LAI)

**FIGURE 5:**
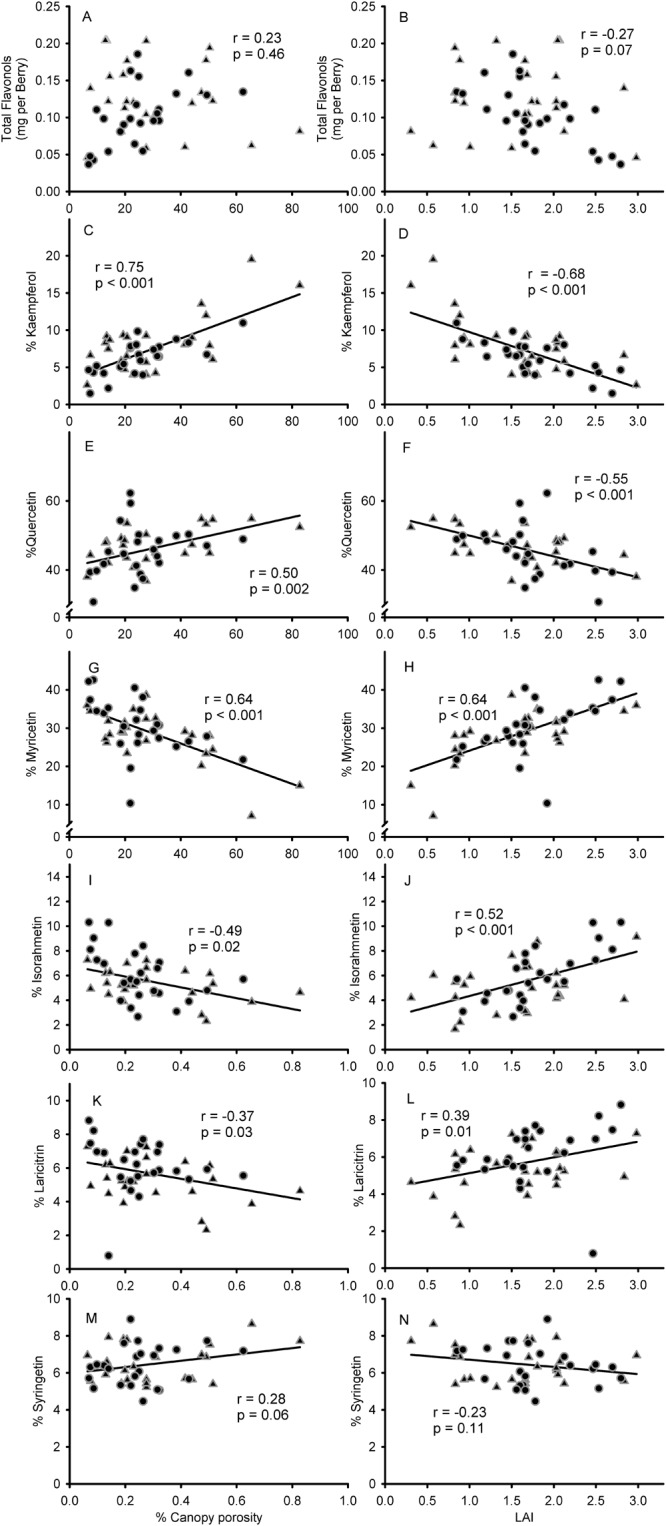
Relationship between estimated canopy porosity and leaf area index (LAI) and flavonol content per berry **(A,B)** % kaempferol **(C,D)**, % quercetin **(E,F)**, % myricetin **(G,H)**, % isorhamnetin **(I,J)**, % laricitin **(K,L)**, and % syringetin **(M,N)** in 5-berry samples collected from the top of individual clusters. Circles for sprawling canopy and triangles for vertical-shoot-positioned trellis. Correlations among all variables recorded available in Supplementary Information 6.

Total flavonols and canopy porosity did not show a significant trend (Figure 5A). Correlations were stronger for the proportion of kaempferol (*r* = 0.75; *p* < 0.001; Figure 5C) compared to quercetin (*r* = 0.50; *p* = 0.002; Figure 5E) and myricetin (*r* = 0.64; *p* < 0.001; Figure 5G) flavonols. LAI, which had an inversely proportional relationship to canopy porosity (*r* = 0.91; *p* < 0.001; data not shown), did not present a significant correlation with total flavonols, either (Figure 5B). Consequently, strong correlations were found with % kaempferol (*r* = -0.68; *p* < 0.001; Figure 5D), % quercetin (*r* = -0.55; *p* < 0.001; Figure 5F) and % myricetin (*r* = 0.64; *p* < 0.001; Figure 5H). Methylated flavonols had slightly weaker correlations with canopy porosity and LAI and in the case of syringetin these were not significant (*r* = 0.28; *p* = 0.06; Figure 5M). % laricitin had similar significant correlations with canopy porosity (*r* = -0.37; *p* = 0.03; Figure 5K) and LAI (*r* = 0.39; *p* = 0.01; Figure 5L) as % myricetin. However, in the case of isorhamnetin and syringetin, their relationships with canopy porosity and LAI were the contrary to their non-methylated homologs Figure 5I,J,M,N) (quercetin and myricetin, respectively).

### Response of Flavonol Content and Profile to Canopy Density, Plant Water Status, and Total Soluble Solids

**FIGURE 6:**
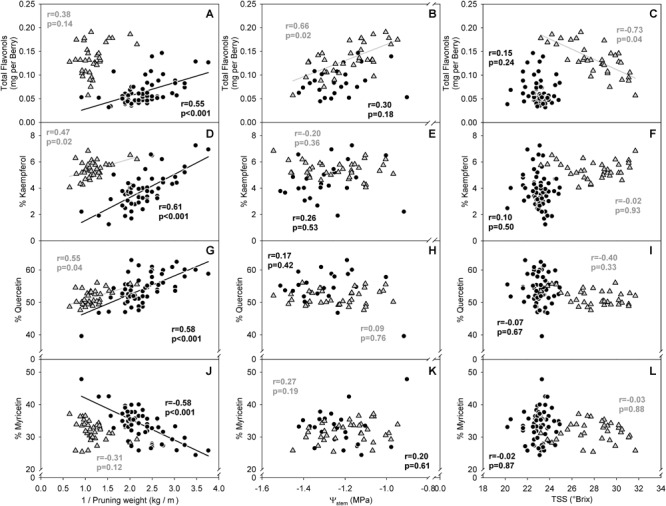
Relationship between flavonol content per berry **(A–C)**, % kaempferol **(D–F)**, % quercetin **(G–I)** and % myricetin **(J–I)** and the inverse of dormant pruning weight **(A,D,G,J)** stem water potential **(B,E,H,K)** and total soluble solids **(C,F,I,L)** of Healdsburg, CA cv. Cabernet Sauvignon trained as double bilateral cordons and sprawling canopy (Gray triangles) and Paso Robles, CA cv. Merlot trained as bilateral cordons and vertical-shoot-positioned trellis (Black circles). Correlations among all variables recorded available in Supplementary Informations 7 and 8.

In the two datasets analyzed (experiment 3), the sensitivity of flavonol content and profiles to canopy size, water status and TSS represented two different wine grape production systems (Table 1). The two sites, Healdsburg, CA and Paso Robles, CA had similar weather conditions during the execution of the study, with very low precipitation and hot temperatures throughout the growing season, which is representative of the coastal viticulture regions of California. Datasets were composed by two different varieties and trellis systems and this most likely affected dormant pruning weights and yields, being obviously greater in Cabernet Sauvignon in Healdsburg, CA which had 2 bilateral cordons and a sprawling canopy versus Merlot in Paso Robles, CA that had one and a vertical trellised canopy. These Cabernet Sauvignon grapes were harvested much riper (23.2–31.7°Brix) than Merlot (20.2–24.9°Brix). The inverse-transformed dormant pruning weights were linearly correlated to flavonol profile but not the flavonol content (Figure 6A,D,G,J). Among these determinations, the % kaempferol had the best coefficients of determination in both Cabernet Sauvignon and Merlot (*r* = 0.47 and *r* = 0.61, respectively). Total flavonol content was strongly correlated with stem water potentials in Healdsburg Cabernet Sauvignon, indicating a reduction of flavonol content with increasing water deficit. However, it must be noted that stem water potentials were also strongly correlated with TSS in Cabernet Sauvignon vineyard (*r* = -0.84; *p* < 0.001; data not shown). Berry TSS only had significant effects on flavonol content in Cabernet Sauvignon. In fact, total flavonol content had a marked decrease with increasing TSS in the Cabernet Sauvignon vineyard (Figure 6C).

**Table 1:** Minima, average, and maxima of yield components, water status, total soluble solids, anthocyanin content, flavonol content and profile, and climatic conditions (from 15th May to 15th October) of two experimental sites used to study variations of vigor, water status and developmental stage on flavonol content and profile (Figure 6).

	Healdsburg, CA, United States,			Paso Robles, CA,		
	Cabernet Sauvignon			United States, Merlot		
	Min	Average	Max	Min	Average	Max
Pruning weights (kg/m)	0.58	1.13	1.94	0.27	0.46	1.10
Yield (kg/m)	0.72	2.69	5.65	0.54	2.24	4.00
Berry weight (g per Berry)	0.68	1.01	1.30	0.84	1.08	1.61
ψ_stem_ (MPa)	–1.45	–1.19	–0.95	–1.42	–1.22	–0.90
TSS (°Brix)	23.2	28.0	31.7	20.2	23.0	24.9
Total anthocyanins (mg per Berry)	1.07	1.98	2.83	1.26	1.70	2.15
Total flavonols (mg per Berry)	0.06	0.13	0.19	0.03	0.06	0.15
% Kaempferol	4.05	5.40	6.83	1.26	3.79	7.25
% Quercetin	47.63	51.5	56.1	39.6	54.0	63.0
% Myricetin	25.4	31.7	37.1	24.4	33.7	47.8
Daily min T (°C)	2.3	8.1	13.3	2.2	8.4	17.1
Daily mean T (°C)	11.0	17.2	23.2	10.5	18.6	29.1
Daily max T (°C)	13.7	28.5	38.2	16.7	29.9	41.2
Daily solar rad. (MJ/m^2^)	2.93	22.23	30.33	5.58	20.86	25.6
Rel humidity (%)	47.3	69.6	95.1	24.8	57.3	91.0

	Total growing season			Total growing season		

Daily precipitations (mm)	51.5			76.9		
Irrigation (L/m)	217.8			672.8		

### Natural Spatial Variability of Grape Light Interception and Effect of Canopy Management Practices

**FIGURE 7:**
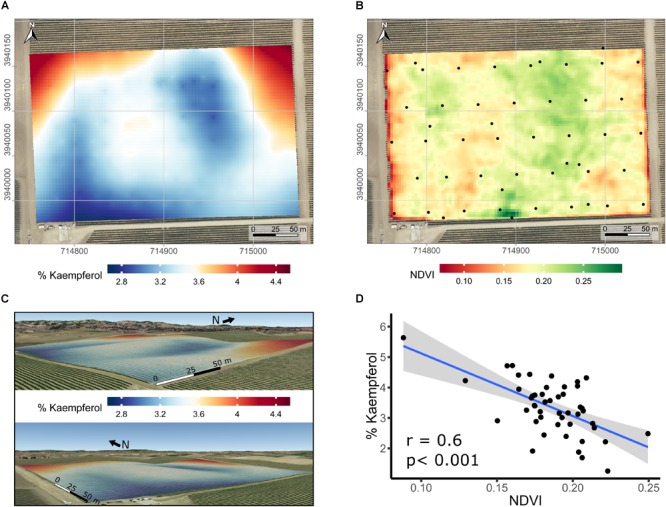
Kriged maps of % kaempferol in 2D **(A)** and 3D **(B)**, maps of satellite-sensed NDVI **(C)**, and relationship between % kaempferol and NDVI **(D)** in a cv. Merlot vineyard trained as bilateral cordons and vertically shoot positioned. **(A)** Root mean squared error associated with the kriging procedure is 0.8% kaempferol estimated with leave-one out cross-validation. Elevation is exaggerated twice in 3D map **(B)** to enhance topographic variation within the vineyard. Coordinates are EPSG:32610. Black dots in the map are the centroid of the 5-vine experimental units. **(B)** Overlap of map in **(A)** on a Google Earth background, view from the South-East (upper) and South-West corner (lower). **(C)** Three meters resolution, satellite-sensed NDVI map dated 2016-07-14. Black dots in map **C** are the centroid of the 5-vine experimental units. Coordinates in **(A,C)** are EPSG:32610, consequently units are in meters. Background map in **(A,C)** is an USGS high resolution aerial image dated 2014-08-11.

Spatial variability in the proportion of kaempferol in flavonols of Merlot was further studied. Spatial interpolation of % kaempferol displayed a distinct spatial pattern based on semi-variogram investigations (data not shown). Because of the relationship between elevation and the proportion of kaempferol, the latter was kriged using universal block kriging with elevation as a covariate (Figure 7A in 2D and Figure 7C in 3D). Including elevation in the geostatistical model improved the results when compared to the ordinary kriging using leave-one-out cross-validation. The cross-validation root mean squared error was 0.8% kaempferol. In addition to the strong correlation between % kaempferol with pruning weights in this vineyard (Figure 6D), % kaempferol had a strong correlation with SAGA wetness index (*r* = -0.48; *p* < 0.001; data not shown) and NDVI (*r* = -0.60; *p* < 0.001; Figure 7B,D). In addition, there was a strong correlation between SAGA wetness index and NDVI (*r* = 0.5; *p* < 0.001; data not shown).

After grouping experimental units by their dormant pruning weights into low, medium and high vigor these came as significantly different from each other (Table 2). These differences in canopy size did not translate into different total flavonol content. Contrarily, the % kaempferol, quercetin and myricetin flavonols was significantly different in the Low vigor group compared to Medium and High. Furthermore, mean difference in these three parameters was greater from Low to Medium than from Medium to High, suggesting an attenuation in the response to increasing canopy size, similar to the relationship reported in Figure 6A.

**Table 2:** Dormant pruning weight and flavonol composition at harvest in a cv. Merlot, Paso Robles, CA, United States, vineyard from vines homogeneously distributed and grouped by their pruning weights and canopy management treatments.

	Pruning wood	Total flavonols
	(kg/m)	(mg per Berry)	% Kaempferol	% Quercetin	% Myricetin
**Vigor groupings (*n* = 15)**
Low	0.39 ± 0.01 c	0.059 ± 0.003	3.92 ± 0.18 a	52 ± 1.1 a	36.8 ± 1.2 b
Medium	0.47 ± 0.01 b	0.05 ± 0.003	3.19 ± 0.21 b	47.6 ± 0.9 b	40.4 ± 1 a
High	0.59 ± 0.04 a	0.049 ± 0.003	2.94 ± 0.23 b	46.6 ± 1.2 b	41.9 ± 1.4 a
*p-*value	<0.001	0.055	0.003	0.009	0.005
**Treatments (*n* = 4)**					
Control	0.47 ± 0.03 a	0.052 ± 0.008 c	3.19 ± 0.18 c	48.5 ± 0.8 c	41.2 ± 0.9 a
Leaf removal	0.45 ± 0.06 ab	0.1 ± 0.004 ab	4.79 ± 0.31 b	51.2 ± 0.4 b	37.3 ± 0.3 b
Shoot thinning	0.34 ± 0.02 b	0.091 ± 0.006 b	5.36 ± 0.41 ab	52.3 ± 1.4 ab	35.4 ± 1.7 b
Combined	0.33 ± 0.03 b	0.135 ± 0.005 a	6.72 ± 0.23 a	55 ± 0.5 a	32.1 ± 0.6 c
*p*-value	0.047	<0.001	<0.001	0.001	<0.001

*Effect of the cultural practices leaf removal, shoot thinning and their combination increasing grape exposure. Means in the same column with no letters in common differ (ANOVA-LSD, *p* < 0.05).*

This expected response was also observed in Merlot grapevine where leaf or shoot removal was applied (Table 2) that had nearly twofold increase in flavonol content and higher % kaempferol and % quercetin in detriment of % myricetin, compared to medium vigor untreated controls. Combining shoot thinning and leaf removal affected % kaempferol by increasing its content greater than when leaf removal was applied alone.

## Discussion

### Flavonol Profile Is Shaped by Solar Radiation Over Time

Regulatory and synthetic genes responsible for flavonol biosynthesis are upregulated by UV-B fraction of solar radiation (Carbonell-Bejerano et al., 2014; Liu et al., 2014), and grape flavonol content increases with exposure to UV-B radiation unequivocally (Berli et al., 2011; Martínez-Lüscher et al., 2014b; Del-Castillo-Alonso et al., 2016b; Matus, 2016). In our study, flavonol content of *V. vinifera* cv. Cabernet Sauvignon under field conditions responded accordingly to increasing solar radiation up to ripeness. However, flavonol content decreased especially in exposed grapes (i.e., 0% shading factor) beyond 22°Brix, reducing the differences between the exposure levels and making total flavonol concentration a poor indicator of solar radiation or canopy architecture. Although reductions in flavonoid content in the weeks prior to harvest are not the rule, they are reported (Castellarin et al., 2007; Bautista-Ortin et al., 2012; Bobeica et al., 2015). Small flavonoid losses may be associated to fruit senescence (the loss of grape skin cellular integrity) in the absence of stress, but exacerbated by high solar radiation and temperature (Mori et al., 2007; Martínez-Lüscher et al., 2017) or severe water stress (Brillante et al., 2017).

The proportion of kaempferol and quercetin were consistently higher in grapes under increased solar radiation (no shade nets, higher canopy porosity and higher vigor). Great similarities can be found in studies reporting kaempferol, quercetin and myricetin percentages (Pastore et al., 2013) and contents (Berli et al., 2011; Del-Castillo-Alonso et al., 2016b). For instance, Del-Castillo-Alonso et al. (2016b) showed a strong reduction (10-fold) of kaempferol-3-O-glucoside when solar UV radiation was screened, similar to decreases quercetin-3-O-glucuronide or quercetin-3-O-glucopyranoside (two and threefold reduction), but much lower than myricetin-3-O-glucoside, which only decreased by 15%. Contrarily to total flavonol content, flavonol profile maintained the differences between shading factors down to the end of the experiment. In addition, the proportion of quercetin and myricetin kept evolving after ripeness (ca. 22°Brix), when net synthesis is presumably not taking place, whereas the proportion of kaempferol stabilized and remained unchanged for a period of several weeks. Flavonol profile constitution under UV-B radiation and water deficit has been described up to ripeness (Martínez-Lüscher et al., 2014a), but not beyond 22°Brix, when flavonols may decrease with increasing TSS (Figure 6C). One plausible reason for profile changing in absence of *de novo* synthesis is a differential degradation of the flavonols according to their substituents in positions 3′ and 5′. The addition of substituents of the flavonoid B-ring may strongly increase the antioxidant capacity of flavonols (Csepregi and Hideg, 2018). This higher antioxidant capacity could explain the differential degradation observed in this study. Under *in vitro* conditions, F3′5′H has been reported to have affinity for flavonols (in addition to dihydroflavonols), which could lead to the conversion of quercetins into myricetins (Kaltenbach et al., 1999).

Overexposed grapes clearly lost a majority of their flavonols (Figure 4D) and other phenolic compounds such as hydroxycynamic acids, gallic acid, flavan-3-ols, anthocyanins and proanthocyanidins (data not shown). Flavonol accumulation is one of the main mechanism of defense against high doses of UV radiation (Agati and Tattini, 2010). However, in studies where high doses of UV-B mimicking ozone depletion were tested (Kakani et al., 2003), no decrease in flavonols with increasing doses were reported. In the present study, where flavonols decreased due to over exposure, grapes received different exposures to full spectrum solar radiation and a subsequent increase in temperature, not only UV-B radiation. Efforts to decouple the effects of solar exposure prove that loss of anthocyanins and flavonols are related to temperature gain rather than the radiation itself (Spayd et al., 2002). In addition, fruit temperatures much lower than the ones recorded in fully exposed clusters in this study (i.e., 46.5°C; Supplementary Information 5) may be responsible for the degradation of flavonoids rather than down regulation of their synthesis (Mori et al., 2007). Under such strong flavonol degradation, % kaempferol continued increasing with solar radiation although at a slightly higher rate when degradation started to take place (Figure 4A,D). However, as cluster temperature is affected by both exposure to solar radiation and air temperature, 550 MJ m^2^ and 11% kaempferol thresholds were found for seeing flavonol degradation. Furthermore, the degradation may be quite sensitive to variations in air temperature during ripening. In fact, 2017 was a hot year at the study site of the experiment with a maximum air temperature of 44°C.

### Influence of Water Status Variability on Flavonol Content and Profile

Flavonoid hydroxylation may be affected by plant water status (Castellarin et al., 2007; Martínez-Lüscher et al., 2014a). In this study, mean Ψ_stem_ had a wide range of variation (-1.42 to -0.90 MPa) and still the correlation between Ψ_stem_ and the proportion of quercetin and myricetin was not significant. One reason for the lack of effect of plant water status may be the overriding effect of solar radiation on flavonol profiles as in previous research water status led only to 10% of change in the proportion of 3′4′5′-OH flavonols, which were mostly myricetin, versus the 40% change induced by UV-B (Martínez-Lüscher et al., 2014a). In addition, although there is a wide range of variation in Ψ_stem_ within the commercial vineyards, these does not include values comparable to a well-watered control (ca. -0.3 MPa) used in field experiments reporting changes in hydroxylation and gene expression (Castellarin et al., 2007). This suggested that higher rates of flavonol hydroxylation in response to water deficit may be taking place by default under the semiarid conditions of our study sites.

### The Use of the Proportion of Kaempferol as a Metabolic Integrator of the Overall Radiation Received by a Grape Berry and Its Implications for Canopy Architecture

The percentage of kaempferol had a strong linear correlation to modeled global radiation. Previous research indicated that total flavonol content and the proportion of kaempferol, quercetin and myricetin changed with exposure to solar radiation (Price et al., 1995; Haselgrove et al., 2000; Pastore et al., 2013; Martínez-Lüscher et al., 2014b; Del-Castillo-Alonso et al., 2016a). However, flavonol content was susceptible to degradation due to solar radiation or over-ripening (Martínez-Lüscher et al., 2017) and the proportion of quercetin and myricetin may be affected by the progress of ripening (Figure 3C,D) or water availability (Martínez-Lüscher et al., 2014a), potentially making of the proportion of kaempferol a better indicator of grape exposure to solar radiation under the conditions and varieties tested. The results presented give the possibility of using flavonol profile for accounting for mean radiation received by a berry. It must be noted that large genotypic differences exist in flavonol profile. For instance, baseline levels of % kaempferol can range from 0% in Tannat to 17% in Muscat Rouge, which are very dark and pale red skinned and have high and low apparent F3′5′H activity, respectively (Mattivi et al., 2006). Therefore, cultivar differences in the slope and intercept of the relationship between radiation-flavonol can be expected. Pastore et al. (2017) reported flavonol profiles of four varieties with changes of different magnitude in the proportion of each flavonol in response to defoliation. However, defoliation always corresponded to an increase in the proportion of kaempferol and quercetin in detriment to myricetin proportion.

A great number of studies have been aiming to assess the effect of solar exposure on, for instance, the aroma composition, organic acids, anthocyanins, and proanthocyanidins (Ryona et al., 2008; Tarara et al., 2008; Chorti et al., 2010; Cohen et al., 2012; Koch et al., 2012). There are different approaches to study the effects of solar radiation on grapes berry including the use of partial (Koch et al., 2012) and complete cluster shading (Downey et al., 2004; Cortell and Kennedy, 2006), canopy management (Matus et al., 2009; Pastore et al., 2013), or random variation among or within cluster exposure to solar radiation (Bergqvist et al., 2001). The use of flavonol profile as a record of overall grape berry exposure allows to study the effect of different levels of solar radiation as a continuous variable, in contrast to studying it as an ordinal variable (i.e., in treatments), to fine-tune the characterization of berries response. In addition, this estimator can be as integrative as required to characterize the microclimate of a single grape berry, clusters, vines or a groups of grapevines.

The robust correlation between the flavonol profile with solar radiation was also useful to verify the changes in canopy architecture. In a vineyard with a great variability in vigor (Table 2 and Figure 7), the amount of solar radiation reaching the grape berry was increased through canopy management practices aiming to increase its exposure to solar radiation. Fruit-zone leaf removal and shoot thinning are the most widely used cultural practices for this purpose, the second being more effective increasing light transmission but potentially reducing yield in the process. Treatments of leaf removal, shoot thinning and their combination, obtained an increase in the proportion of kaempferol and quercetin in detriment of myricetin in the flavonol profile of Merlot due to greater grape berry exposure to solar radiation. Increasing the exposure of grape berry and vegetation vigor control are key factors in the production of quality grapes, such as anthocyanins and tannins accumulation and the removal of herbaceous aromas (Ristic et al., 2007; Matus et al., 2009; Koch et al., 2012). In addition, more open canopies are associated with higher wind speed and lower relative humidity at the fruit-zone, reducing the incidence of fungal rot (English et al., 1993).

The exposure of a single cluster was indicative of the local estimations of canopy architecture parameters, canopy porosity and LAI. These are relevant ecophysiological parameters as they are related light interception efficiency (Oyarzun et al., 2007), but also estimated LAI, can be used to model carbon assimilation or evapotranspiration in plant communities when the scale is too large to take actual measurements of leaf area (Gan et al., 2018). Flavonol profile was also related to satellite NDVI, a widely used remote sensing estimator of canopy size based on the specific spectral reflectance of chlorophyll tissues respect to the ground. In turn, % kaempferol, dormant pruning weights and NDVI were related to site hydrology (SAGA wetness index), suggesting that gradients in potential plant water availability within the vineyard may have led to differences in vigor, and thus, canopy architecture and berry exposure to solar radiation. This variability patterns have been observed by others, associating adequate canopy size with improved grape composition (Baluja et al., 2012; Scarlett et al., 2014).

## Conclusion

This study aimed make more accessible the determination of flavonol profiles in anthocyanin-rich tissues by fine-tuning available HPLC-DAD methods. We also aimed to propose flavonol profile, and specially the proportion of kaempferol, as a validation tool for the accumulated solar radiation received by grapes and test its reliability and use. In our results, flavonol profile was strongly related to canopy porosity and LAI estimations for individual clusters and to dormant pruning weight at the vine level. This reliability was based on the stability of % kaempferol through the progress of ripening and increasing water deficit. The assessment of berry exposure was exemplified by the response of flavonol profile to vigor groupings and to cultural practices aiming to increase the exposure of the grapes or balancing vine vigor. Given the extended use of HPLC-DAD to determine anthocyanin profile in research, we provided the principles to determine and understand flavonol profile constitution with this method. The flavonol profile is proposed as a fine indicator of the solar radiation intercepted and accumulated by berries and useful to discuss the effect of solar radiation or canopy architecture on grape composition.

## Author Contributions

JM-L contributed to the data acquisition and elaborated the manuscript draft and the final version. LB contributed to the data acquisition, performed the spatial analyses, and reviewed the manuscript. SK contributed to the data acquisition, led the projects yielding the data, and reviewed the manuscript.

## Conflict of Interest Statement

The authors declare that the research was conducted in the absence of any commercial or financial relationships that could be construed as a potential conflict of interest.

## References

[B1] Agati G. Brunetti C. Di Ferdinando M. Ferrini F. Pollastri S. Tattini M. (2013). Functional roles of flavonoids in photoprotection: new evidence, lessons from the past. *Plant Physiol. Biochem.* 72 35–45. 10.1016/j.plaphy.2013.03.014 23583204

[B2] Agati G. Tattini M. (2010). Multiple functional roles of flavonoids in photoprotection. *New Phytol.* 186 786–793. 10.1111/j.1469-8137.2010.03269.x 20569414

[B3] Baluja J. Diago M. P. Goovaerts P. Tardaguila J. (2012). Assessment of the spatial variability of anthocyanins in grapes using a fluorescence sensor: relationships with vine vigour and yield. *Precis. Agric.* 13 457–472. 10.1007/s11119-012-9261-x

[B4] Bautista-Ortin A. B. Rodriguez-Rodriguez P. Gil-Munoz R. Jimenez-Pascual E. Busse-Valverde N. Martinez-Cutillas A. (2012). Influence of berry ripeness on concentration, qualitative composition and extractability of grape seed tannins. *Aust. J. Grape Wine Res.* 18 123–130. 10.1111/j.1755-0238.2012.00178.x

[B5] Bergqvist J. Dokoozlian N. Ebisuda N. (2001). Sunlight exposure and temperature effects on berry growth and composition of cabernet sauvignon and grenache in the central san joaquin valley of California. *Am. J. Enol. Vitic.* 52 1–7.

[B6] Berli F. J. Fanzone M. Piccoli P. Bottini R. (2011). Solar UV-B and ABA are involved in phenol metabolism of Vitis vinifera L. increasing biosynthesis of berry skin polyphenols. *J. Agric. Food Chem.* 59 4874–4884. 10.1021/jf200040z 21469737

[B7] Bianchi S. Cahalan C. Hale S. Gibbons J. M. Michael J. (2017). Rapid assessment of forest canopy and light regime using smartphone hemispherical photography. *Ecol. Evol.* 7 10556–10566. 10.1002/ece3.3567 29299237 PMC5743530

[B8] Bobeica N. Poni S. Hilbert G. Renaud C. Gomès E. Delrot S. (2015). Differential responses of sugar, organic acids and anthocyanins to source-sink modulation in cabernet sauvignon and sangiovese grapevines. *Front. Plant Sci.* 6:382. 10.3389/fpls.2015.00382 26074942 PMC4448006

[B9] Brillante L. Martínez-Luscher J. Yu R. Plank C. M. C. M. Sanchez L. Bates T. L. (2017). Assessing spatial variability of grape skin flavonoids at the vineyard scale based on plant water status mapping. *J. Agric. Food Chem.* 65 5255–5265. 10.1021/acs.jafc.7b01749 28602091

[B10] Carbonell-Bejerano P. Diago M. P. Martínez-Abaigar J. Martínez-Zapater J. M. Tardáguila J. Núñez-Olivera E. (2014). Solar ultraviolet radiation is necessary to enhance grapevine fruit ripening transcriptional and phenolic responses. *BMC Plant Biol.* 14:183. 10.1186/1471-2229-14-183 25012688 PMC4099137

[B11] Castagna A. Csepregi K. Neugart S. Zipoli G. Veèeøová K. Jakab G. (2017). Environmental plasticity of Pinot noir grapevine leaves: a trans-European study of morphological and biochemical changes along a 1,500-km latitudinal climatic gradient. *Plant. Cell Environ.* 40 2790–2805. 10.1111/pce.13054 28792065

[B12] Castellarin S. D. Di Gaspero G. (2007). Transcriptional control of anthocyanin biosynthetic genes in extreme phenotypes for berry pigmentation of naturally occurring grapevines. *BMC Plant Biol.* 7:46. 10.1186/1471-2229-7-46 17760970 PMC2147006

[B13] Castellarin S. D. Pfeiffer A. Silivotti P. Degan M. Peterlunger E. Di Gaspero G. (2007). Transcriptional regulation of anthocyanin biosynthesis in ripening fruits of grapevine under seasonal water deficit. *Plant. Cell Environ.* 30 1381–1399. 10.1111/j.1365-3040.2007.01716.x 17897409

[B14] Chorti E. Guidoni S. Ferrandino A. Novello V. (2010). Effect of different cluster sunlight exposure levels on ripening and anthocyanin accumulation in Nebbiolo grapes. *Am. J. Enol. Vitic.* 61 23–30.

[B15] Cohen S. D. Tarara J. M. Gambetta G. A. Matthews M. A. Kennedy J. A. (2012). Impact of diurnal temperature variation on grape berry development, proanthocyanidin accumulation, and the expression of flavonoid pathway genes. *J. Exp. Bot.* 63 2655–2665. 10.1093/jxb/err449 22268158 PMC3346226

[B16] Conrad O. Bechtel B. Bock M. Dietrich H. Fischer E. Gerlitz L. (2015). System for automated geoscientific analyses (SAGA) v. 2.1. 4. *Geosci. Model Dev.* 8 1991–2007. 10.5194/gmd-8-1991-2015

[B17] Cooper J. E. (2004). Multiple responses of rhizobia to flavonoids during legume root infection. *Adv. Bot. Res.* 41 1–62. 10.1016/S0065-2296(04)41001-5

[B18] Cortell J. M. Kennedy J. A. (2006). Effect of shading on accumulation of flavonoid compounds in (Vitis vinifera L.) pinot noir fruit and extraction in a model system. *J. Agric. Food Chem.* 54 8510–8520. 10.1021/jf0616560 17061828

[B19] Csepregi K. Hideg É. (2018). Phenolic compound diversity explored in the context of photo-oxidative stress protection. *Phytochem. Anal.* 29 129–136. 10.1002/pca.2720. 28895264

[B20] Czemmel S. Stracke R. Weisshaar B. Cordon N. Harris N. N. Walker A. R. (2009). The grapevine R2R3-MYB transcription factor VvMYBF1 regulates flavonol synthesis in developing grape berries. *Plant Physiol.* 151 1513–1530. 10.1104/pp.109.142059 19741049 PMC2773091

[B21] Dai Z. W. Meddar M. Renaud C. Merlin I. Hilbert G. Delrot S. (2014). Long-term *in vitro* culture of grape berries and its application to assess the effects of sugar supply on anthocyanin accumulation. *J. Exp. Bot.* 65 4665–4677. 10.1093/jxb/ert489 24477640 PMC4115254

[B22] de Mendiburu M. F. (2016). *Package ‘Agricolae.’ Statistical Procedures for Agricultural Research. Version 1.3-0*.

[B23] Del-Castillo-Alonso M. Á. Castagna A. Csepregi K. Hideg É. Jakab G. Jansen M. A. K. (2016a). Environmental factors correlated with the metabolite profile of Vitis vinifera cv. Pinot Noir berry skins along a European latitudinal gradient. *J. Agric. Food Chem.* 64 8722–8734. 10.1021/acs.jafc.6b03272. 27794599

[B24] Del-Castillo-Alonso M. Á. Diago M. P. Tomás-Las-Heras R. Monforte L. Soriano G. Martínez-Abaigar J. (2016b). Effects of ambient solar UV radiation on grapevine leaf physiology and berry phenolic composition along one entire season under mediterranean field conditions. *Plant Physiol. Biochem.* 109 374–386. 10.1016/j.plaphy.2016.10.018. 27810677

[B25] Dixon R. A. Liu C. Jun J. H. (2013). Metabolic engineering of anthocyanins and condensed tannins in plants. *Curr. Opin. Biotechnol.* 24 329–335. 10.1016/j.copbio.2012.07.004 22901316

[B26] Downey M. O. Harvey J. S. Robinson S. P. (2004). The effect of bunch shading on berry development and flavonoid accumulation in Shiraz grapes. *Aust. J. Grape Wine Res.* 10 55–73. 10.1111/j.1755-0238.2004.tb00008.x

[B27] Downey M. O. Rochfort S. (2008). Simultaneous separation by reversed-phase high-performance liquid chromatography and mass spectral identification of anthocyanins and flavonols in Shiraz grape skin. *J. Chromatogr. A* 1201 43–47. 10.1016/j.chroma.2008.06.002 18573501

[B28] English J. T. Kaps M. L. Moore J. F. Hill J. Nakova M. (1993). Leaf removal for control of botrytis bunch rot of wine grapes in the midwestern United States. *Plant Dis.* 77 1224–1227. 10.1094/PD-77-1224

[B29] Falginella L. Castellarin S. D. Testolin R. Gambetta G. A. Morgante M. Di Gaspero G. (2010). Expansion and subfunctionalisation of flavonoid 3’,5’-hydroxylases in the grapevine lineage. *BMC Genomics* 11:562. 10.1186/1471-2164-11-562 20939908 PMC3091711

[B30] Fournier-Level A. Hugueney P. Verries C. This P. Ageorges A. (2011). Genetic mechanisms underlying the methylation level of anthocyanins in grape (Vitis vinifera L.). *BMC Plant Biol.* 11:179. 10.1186/1471-2229-11-179 22171701 PMC3264682

[B31] Gan R. Zhang Y. Shi H. Yang Y. Eamus D. Cheng L. (2018). Use of satellite leaf area index estimating evapotranspiration and gross assimilation for Australian ecosystems. *Ecohydrology* 11:e1974 10.1002/eco.1974

[B32] Haselgrove L. Botting D. van Heeswijck R. Høj P. B. Dry P. R. Ford C. (2000). Canopy microclimate and berry composition: the effect of bunch exposure on the phenolic composition of Vitis vinifera L cv. Shiraz grape berries. *Aust. J. Grape Wine Res.* 6 141–149. 10.1111/j.1755-0238.2000.tb00173.x

[B33] Hermosín-Gutiérrez I. Castillo-Muñoz N. Gómez-Alonso S. García-Romero E. (2011). “Flavonol profiles for grape and wine authentication,” in *Progress in Authentication of Food and Wine* eds Ebeler S. E. Winterhalter G. R. Takeoka P. (Olympia, WA: American Chemical Society).

[B34] Hilbert G. Temsamani H. Bordenave L. Pedrot E. Chaher N. Cluzet S. (2015). Flavonol profiles in berries of wild Vitis accessions using liquid chromatography coupled to mass spectrometry and nuclear magnetic resonance spectrometry. *Food Chem.* 169 49–58. 10.1016/j.foodchem.2014.07.079 25236197

[B35] Kakani V. G. Reddy K. R. Zhao D. Sailaja K. (2003). Field crop responses to ultraviolet-B radiation: a review. *Agric. For. Meteorol.* 120 191–218. 10.1016/j.agrformet.2003.08.015 24725638

[B36] Kaltenbach M. Schröder G. Schmelzer E. Lutz V. Schröder J. (1999). Flavonoid hydroxylase from *Catharanthus roseus*: cDNA, heterologous expression, enzyme properties and cell-type specific expression in plants. *Plant J.* 19 183–193. 10.1046/j.1365-313X.1999.00524.x 10476065

[B37] Koch A. Ebeler S. E. Williams L. E. Matthews M. A. (2012). Fruit ripening in Vitis vinifera: light intensity before and not during ripening determines the concentration of 2-methoxy-3-isobutylpyrazine in cabernet sauvignon berries. *Physiol. Plant.* 145 275–285. 10.1111/j.1399-3054.2012.01572.x 22224579

[B38] Koes R. E. Quattrocchio F. Mol J. N. M. (1994). The flavonoid biosynthetic pathway in plants: function and evolution. *BioEssays* 16 123–132. 10.1002/bies.950160209

[B39] Kong C. Xu X. Zhou B. Hu F. Zhang C. Zhang M. (2004). Two compounds from allelopathic rice accession and their inhibitory activity on weeds fungal pathogens. *Phytochemistry* 65 1123–1128. 10.1016/j.phytochem.2004.02.017 15110693

[B40] Kolb C. A. Käser M. A. Kopecký J. Zotz G. Riederer M. Pfündel E. E. (2001). Effects of natural intensities of visible and ultraviolet radiation on epidermal ultraviolet screening and photosynthesis in grape leaves. *Plant Physiol.* 127 863–875. 10.1104/pp.010373 11706169 PMC129258

[B41] Liu L. Gregan S. Winefield C. Jordan B. (2014). From UVR8 to flavonol synthase: UV-B-induced gene expression in Sauvignon blanc grape berry. *Plant Cell Environ.* 38 905–919. 10.1111/pce.12349 24738597

[B42] Malacarne G. Costantini L. Coller E. Battilana J. Velasco R. Vrhovsek U. (2015). Regulation of flavonol content and composition in (Syrah × Pinot Noir) mature grapes: integration of transcriptional profiling and metabolic quantitative trait locus analyses. *J. Exp. Bot.* 66 4441–4453. 10.1093/jxb/erv243 26071529 PMC4507773

[B43] Martínez-Lüscher J. Chen C. C. L. Brillante L. Kurtural S. K. (2017). Partial solar radiation exclusion with color shade nets reduce the degradation of organic acids and flavonoids of grape berry (Vitis vinifera L.). *J. Agric. Food Chem.* 65 10693–10702. 10.1021/acs.jafc.7b04163 29141407

[B44] Martínez-Lüscher J. Sánchez-Díaz M. Delrot S. Aguirreolea J. Pascual I. Gomès E. (2014a). Ultraviolet-B radiation and water deficit interact to alter flavonol and anthocyanin profile in grapevine berries through transcriptomic regulation. *Plant Cell Physiol.* 55 1925–1936. 10.1093/pcp/pcu121 25231967

[B45] Martínez-Lüscher J. Torres N. Hilbert G. Richard T. Sánchez-Díaz M. Delrot S. (2014b). Ultraviolet-B radiation modifies the quantitative and qualitative profile of flavonoids and amino acids in grape berries. *Phytochemistry* 102 106–114. 10.1016/j.phytochem.2014.03.014 24713570

[B46] Mattivi F. Guzzon R. Vrhovsek U. Stefanini M. Velasco R. (2006). Metabolite profiling of grape: flavonols and anthocyanins. *J. Agric. Food Chem.* 54 7692–7702. 10.1021/jf061538c 17002441

[B47] Matus J. T. (2016). Transcriptomic and metabolomic networks in the grape berry illustrate that it takes more than flavonoids to fight against ultraviolet radiation. *Front. Plant Sci.* 7:1337. 10.3389/fpls.2016.01337 27625679 PMC5003916

[B48] Matus J. T. Cavallini E. Loyola R. Höll J. Finezzo L. Dal Santo S. (2017). A group of grapevine MYBA transcription factors located in chromosome 14 control anthocyanin synthesis in vegetative organs with different specificities compared with the berry color locus. *Plant J.* 91 220–236. 10.1111/tpj.13558 28370629

[B49] Matus J. T. Loyola R. Vega A. Pena-Neira A. Bordeu E. Arce-Johnson P. (2009). Post-veraison sunlight exposure induces MYB-mediated transcriptional regulation of anthocyanin and flavonol synthesis in berry skins of Vitis vinifera. *J. Exp. Bot.* 60 853–867. 10.1093/jxb/ern336 19129169 PMC2652055

[B50] Mori K. Goto-Yamamoto N. Kitayama M. Hashizume K. (2007). Loss of anthocyanins in red-wine grape under high temperature. *J. Exp. Bot.* 58 1935–1945. 10.1093/jxb/erm055 17452755

[B51] Muggeo V. M. R. (2008). Segmented: an r package to fit regression models with broken-line relationships. *R News* 8 20–25.

[B52] Nakabayashi R. Yonekura-Sakakibara K. Urano K. Suzuki M. Yamada Y. Nishizawa T. (2014). Enhancement of oxidative and drought tolerance in Arabidopsis by overaccumulation of antioxidant flavonoids. *Plant J.* 77 367–379. 10.1111/tpj.12388 24274116 PMC4282528

[B53] Osorio F. Vallejos R. Cuevas F. (2014). *SpatialPack: Package for Analysis of Spatial Data. R Packag. version 0.2-3*. Available at: R-project. org/package=Spat

[B54] Oyarzun R. A. Stöckle C. O. Whiting M. D. (2007). A simple approach to modeling radiation interception by fruit-tree orchards. *Agric. For. Meteorol.* 142 12–24. 10.1016/j.agrformet.2006.10.004

[B55] Pastore C. Allegro G. Valentini G. Muzzi E. Filippetti I. (2017). Anthocyanin and flavonol composition response to veraison leaf removal on cabernet sauvignon. Nero d’avola, raboso piave and sangiovese Vitis vinifera L. cultivars. *Sci. Hortic.* 218 147–155. 10.1016/j.scienta.2017.01.048

[B56] Pastore C. Zenoni S. Fasoli M. Pezzotti M. Tornielli G. B. Filippetti I. (2013). Selective defoliation affects plant growth, fruit transcriptional ripening program and flavonoid metabolism in grapevine. *BMC Plant Biol.* 13:30. 10.1186/1471-2229-13-30 23433030 PMC3599245

[B57] Pebesma E. J. (2004). Multivariable geostatistics in S: the gstat package. *Comput. Geosci.* 30 683–691. 10.1016/j.cageo.2004.03.012

[B58] Price S. F. Breen P. J. Valladao M. Watson B. T. (1995). Cluster sun exposure and quercetin in pinot noir grapes and wine. *Am. J. Enol. Vitic.* 46 187–194.

[B59] Riaño D. Chuvieco E. Salas J. Aguado I. (2003). Assessment of different topographic corrections in landsat-TM data for mapping vegetation types. *IEEE Trans. Geosci. Remote Sens.* 41 1056–1061. 10.1109/TGRS.2003.811693

[B60] Ristic R. Downey M. O. Iland P. G. Bindon K. Francis I. L. Herderich M. (2007). Exclusion of sunlight from Shiraz grapes alters wine colour, tannin and sensory properties. *Aust. J. Grape Wine Res.* 13 53–65. 10.1111/j.1755-0238.2007.tb00235.x

[B61] Ryona I. Pan B. S. Intrigliolo D. S. Lakso A. N. Sacks G. L. (2008). Effects of cluster light exposure on 3-Isobutyl-2-methoxypyrazine accumulation and degradation patterns in red wine grapes (Vitis vinifera L. Cv. Cabernet Franc). *J. Agric. Food Chem.* 56 10838–10846. 10.1021/jf801877y 18942833

[B62] Scarlett N. J. Bramley R. G. V. Siebert T. E. (2014). Within-vineyard variation in the ‘pepper’compound rotundone is spatially structured and related to variation in the land underlying the vineyard. *Aust. J. Grape Wine Res.* 20 214–222. 10.1111/ajgw.12075

[B63] Spayd S. E. Tarara J. M. Mee D. L. Ferguson J. C. (2002). Separation of sunlight and temperature effects on the composition of Vitis vinifera cv. Merlot berries. *Am. J. Enol. Vitic.* 53:3.

[B64] Tarara J. M. Lee J. Spayd S. E. Scagel C. F. (2008). Berry temperature and solar radiation alter acylation, proportion, and concentration of anthocyanin in Merlot grapes. *Am. J. Enol. Vitic.* 59 235–247.

[B65] ter Steege H. (1997). *WINPHOT, a Programme to Analyze Vegetation Indices, Light and Light Quality from Hemispherical Photographs*. Utrecht: Utrecht University.

[B66] Welles J. M. Norman J. M. (1991). Instrument for indirect measurement of canopy architecture. *Agron. J.* 83 818–825. 10.2134/agronj1991.00021962008300050009x

