# Supplementary material for: Flavonol Profile Is a Reliable Indicator to Assess Canopy Architecture and the Exposure of Red Wine Grapes to Solar Radiation
Source: Front Plant Sci. 2019 Jan 31;10:10. doi: 10.3389/fpls.2019.00010 (PMC6365461; doi:10.3389/fpls.2019.00010)
Supplement: Supplementary file 1 [file Table_1.docx]

Supplementary Information


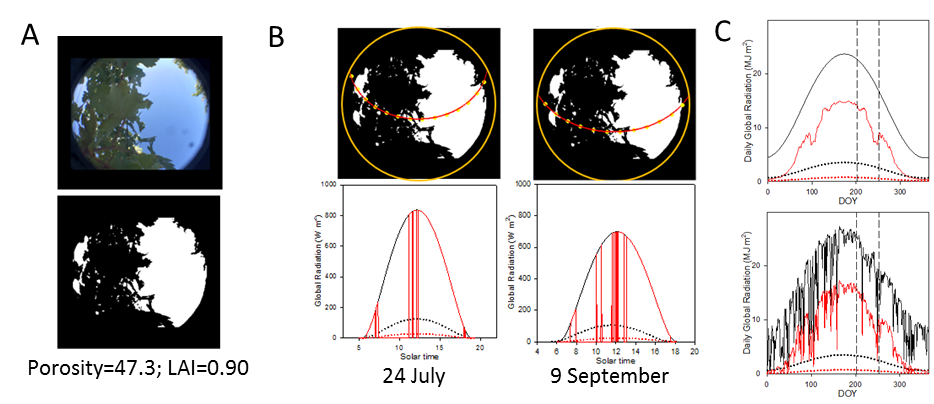


**Additional Information 1 (Figure).** Steps to estimate canopy porosity, LAI and global radiation at the cluster level using 150° lens image of the canopy from the cluster perspective. A, Raw image and thresholded blue channel was used to calculate canopy porosity and plant area index. B, Trajectory of the sun at and modelled global radiation from beginning of ripening (24 July) through harvest (9 September). Yellow lines represent the predicted margins of the image if 180° lens were used. C, Modelled daily accumulated global radiation above and under the canopy and corrected values using measured data from the onsite CIMIS meteorological station. Black lines represent direct global radiation above the canopy and red lines represent global radiation under the canopy. Likewise, dotted lines represent diffuse light.


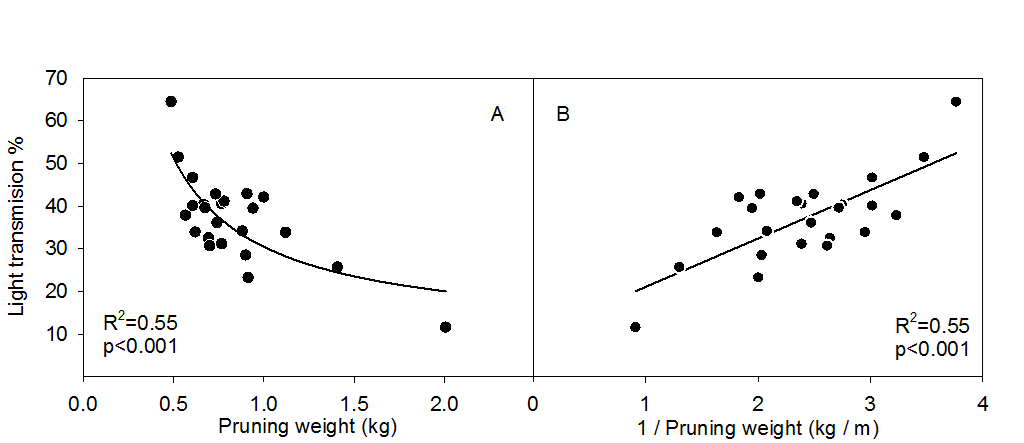


**Supplementary Information 2 (Figure).** Extinction of light by increasing canopy density (dormant pruning weight; A) and linear relationship between light transmission and the inverse of dormant pruning weights per meter of row (B)
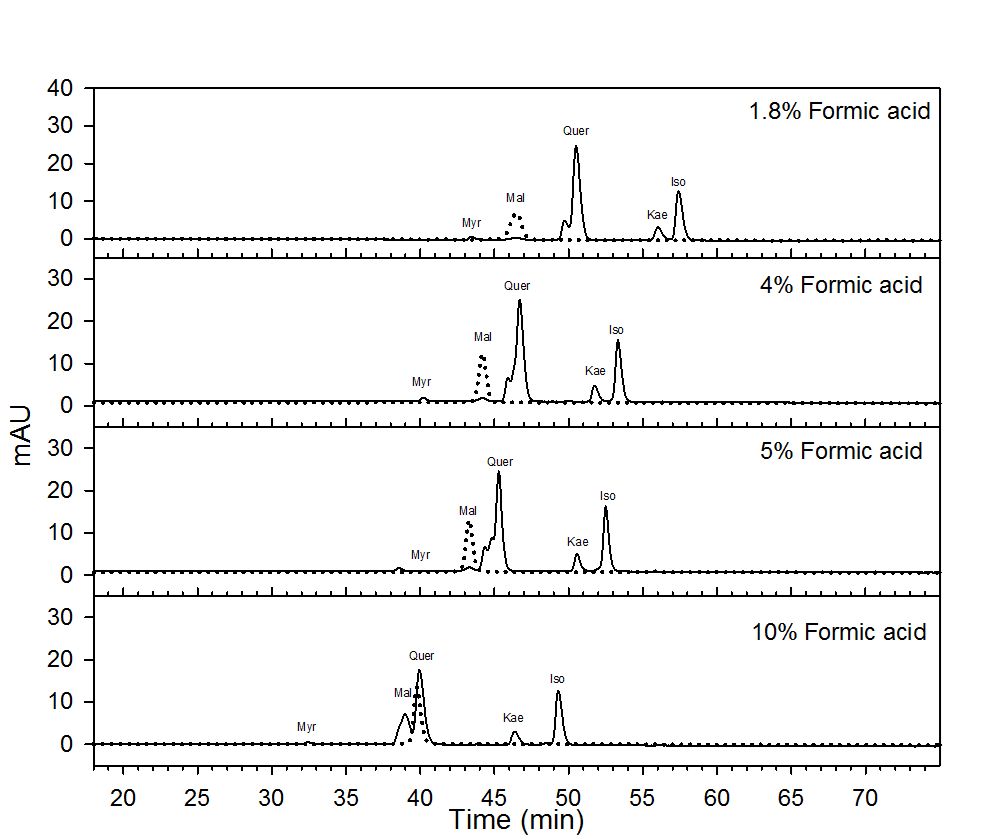


**Supplementary Information 3 (Figure)**. Effect of formic acid concentration on the HPLC retention times of flavonols (solid) commercial standards and Malvidin-3-*o*-glc (dotted).

**Supplementary Information 5 (Figure)**. Cluster temperatures measured with an infrared thermometer on fully exposed clusters in a Cabernet sauvignon vineyard with vertical-shoot-position trellis with NE to SW row orientation in Oakville, CA on 11 September 2017.


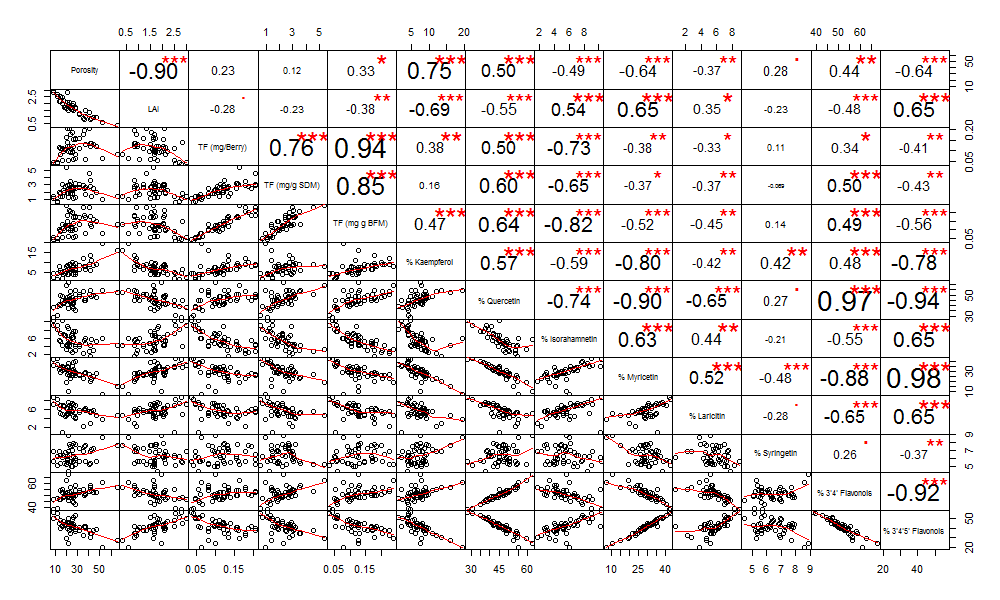


**Supplementary Information 6**. Paired Pearson’s correlations and local regression of Inverse pruning dormant weights (kg^-1^), stem water potentials, total soluble solids, total flavonols (per berry, per gram of dried skin and per gram of berry fresh mass), % kaempferol, % quecetin, % isorhamnetin, % myricetin, %laricitin, % syringetin, % 3’4’ Flavonols and % 3’4’5’ Flavonols in experiment 2.


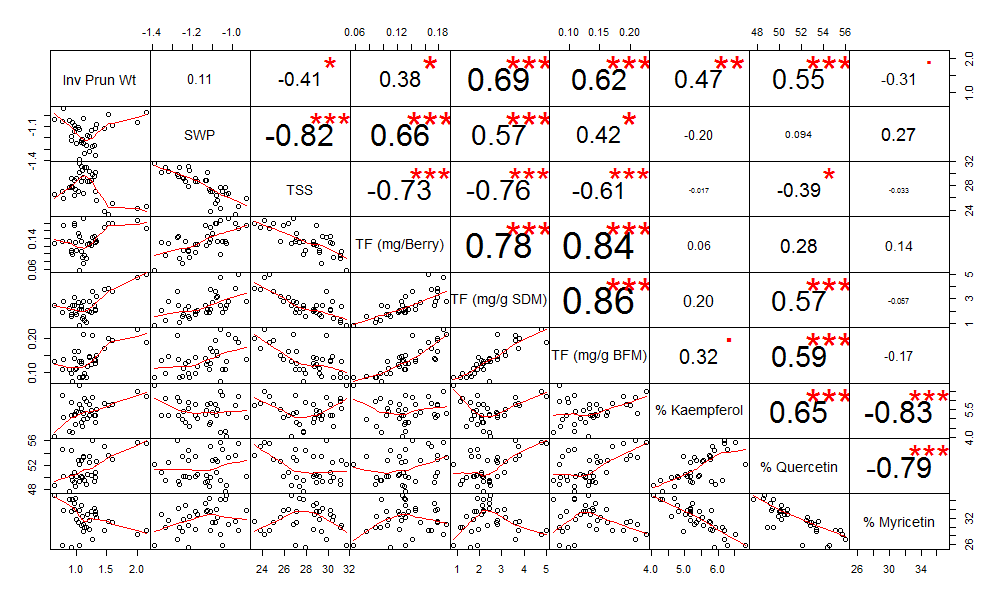


**Supplementary Information 7**. Paired Pearson’s correlations (non-spatial analyses) and local regression of Inverse pruning dormant weights (kg^-1^), stem water potentials, total soluble solids, total flavonols (per berry, per gram of dried skin and per gram of berry fresh mass), % kaempferol, % quecetin and % myricetin in experiment 3: Healdsburg, CA cv. Cabernet Sauvignon trained as double bilateral cordons and sprawling canopy.


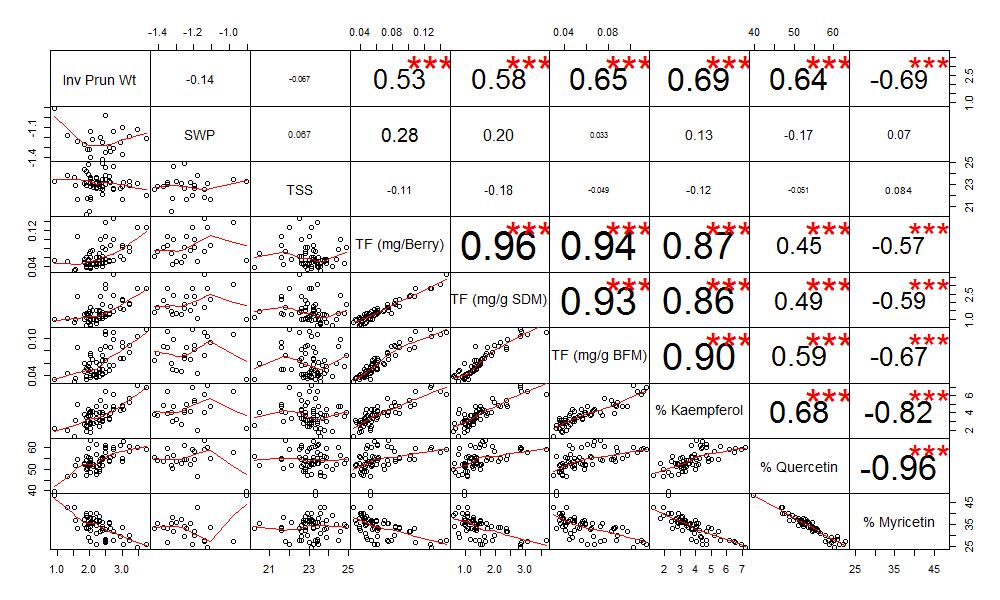


**Supplementary Information 8**. Paired Pearson’s correlations (non-spatial analyses) and local regression of Inverse pruning dormant weights (kg^-1^), stem water potentials, total soluble solids, total flavonols (per berry, per gram of dried skin and per gram of berry fresh mass), % kaempferol, % quercetin and % myricetin in experiment 3: Paso Robles, CA cv. Merlot trained as bilateral cordons and vertical-shoot-positioned trellis.
