# Supplementary material for: Flavonol Profile Is a Reliable Indicator to Assess Canopy Architecture and the Exposure of Red Wine Grapes to Solar Radiation
Source: Front Plant Sci. 2019 Jan 31;10:10. doi: 10.3389/fpls.2019.00010 (PMC6365461; doi:10.3389/fpls.2019.00010)
Supplement: Supplementary file 2 [file Data_Sheet_1.pdf]

Supplementary Information 4

Gradient for 2% HCOOH

	Target % (V/V)			Gradient	
				% Phase A	% Phase B
Time (min)	% ACN	% HCOOH	% H_2O	(2% HCOOH in H_2O)	(2% HCOOH in ACN)
0	8	2	90	91.8	8.2
8	8	2	90	91.8	8.2
25	12.2	2	85.8	87.6	12.4
35	16.9	2	81.1	82.8	17.2
70	35.7	2	62.3	63.6	36.4
70	65	2	33	33.7	66.3
75	65	2	33	33.7	66.3
80	8	2	90	91.8	8.2
90	8	2	90	91.8	8.2

Gradient for 4% HCOOH

	Target % (V/V)			Gradient	
				% Phase A	% Phase B
Time (min)	% ACN	% HCOOH	% H_2O	(4% HCOOH in H_2O)	(4% HCOOH in ACN)
0	8	4	88	91.7	8.3
8	8	4	88	91.7	8.3
25	12.2	4	83.8	87.3	12.7
35	16.9	4	79.1	82.4	17.6
70	35.7	4	60.3	62.8	37.2
70	65	4	31	32.3	67.7
75	65	4	31	32.3	67.7
80	8	4	88	91.7	8.3
90	8	4	88	91.7	8.3

Gradient for 5% HCOOH

	Target % (V/V)			Gradient	
				% Phase A	% Phase B
Time (min)	% ACN	% HCOOH	% H_2O	(5% HCOOH in H_2O)	(5% HCOOH in ACN)
0	8	5	87	91.6	8.4
8	8	5	87	91.6	8.4
25	12.2	5	82.8	87.2	12.8
35	16.9	5	78.1	82.2	17.8
70	35.7	5	59.3	62.4	37.6
70	65	5	30	31.6	68.4
75	65	5	30	31.6	68.4
80	8	5	87	91.6	8.4
90	8	5	87	91.6	8.4

Gradient for 10% HCOOH

	Target % (V/V)			Gradient	
				% Phase A	% Phase B
Time (min)	% ACN	% HCOOH	% H_2O	(10% HCOOH in H_2O)	(10% HCOOH in ACN)
0	8	10	82	91.1	8.9
8	8	10	82	91.1	8.9
25	12.2	10	77.8	86.4	13.6
35	16.9	10	73.1	81.2	18.8
70	35.7	10	54.3	60.3	39.7
70	65	10	25	27.8	72.2
75	65	10	25	27.8	72.2
80	8	10	82	91.1	8.9
90	8	10	82	91.1	8.9
